# Supplementary material for: Youth Exposure to Hate in the Online Space: An Exploratory Analysis
Source: Int J Environ Res Public Health. 2020 Nov 17;17(22):8531. doi: 10.3390/ijerph17228531 (PMC7698507; doi:10.3390/ijerph17228531)
Supplement: Supplementary file 1 [file ijerph-17-08531-s001.pdf]

If you intend to use this questionnaire for your project, please cite the publication and inform the authors by sending an e-mail to preparedness@hsph.harvard.edu

1. Select which grade you were enrolled in as of September 2018:
*Check **only one** box.*

- ☐ 8th grade
- ☐ 9th grade
- ☐ 10th grade
- ☐ 11th grade
- ☐ 12th grade

2. During the last year, what have most of your grades been up to now? *Check **only one** box.*

- ☐ A
- ☐ A-, B+
- ☐ B
- ☐ B-, C+
- ☐ C or lower

3. What is your age? *Check **only one** box.*

- ☐ 14
- ☐ 15
- ☐ 16
- ☐ 17
- ☐ 18
- ☐ 19
- ☐ Over 19

4. What gender do you identify with? *Check **only one** box.*

- ☐ Male
- ☐ Female
- ☐ Rather not say
- ☐ Both

5. What race/ethnicity do you consider yourself? *Check **all boxes** that apply – multiple answers are allowed*

- ☐ White
- ☐ American Indian or Alaska Native
- ☐ Asian
- ☐ Black or African American
- ☐ Hispanic or Latino
- ☐ Native Hawaiian or other Pacific Islander
- ☐ Cape Verdean
- ☐ Haitian
- ☐ Don't know
- ☐ Rather not say
- ☐ Other (please specify below)

____

6. **How much time do you, on average, spend using technology each day NOT including for schoolwork (phone, gaming system, computer, tablet (iPad), etc.)?** *Check **only one** box.*
   - ☐ Less than 1 hour
   - ☐ More than 1 but less than 3 hours
   - ☐ More than 3 but less than 6 hours
   - ☐ More than 6 hours

7. **How much do you agree with the following statements?** *For each statement (a through k) below, indicate how much you agree with the statement by CIRCLING the most appropriate answer, Disagree, Somewhat Agree, Agree or I am not sure.*

a. It is easier for me to connect with others online than talking in person	Disagree	I am not sure	Somewhat Agree	Agree
b. While online I can express my true feelings and thoughts	Disagree	I am not sure	Somewhat Agree	Agree
c. While online I like that I can interact with people without telling them who I am	Disagree	I am not sure	Somewhat Agree	Agree
d. It is easier for me to write things online that would be hard to say in person	Disagree	I am not sure	Somewhat Agree	Agree
e. It is easier for me to communicate online because I can reply to a message only if I want to	Disagree	I am not sure	Somewhat Agree	Agree
f. I like to create an image in my head of the other person when I read their e-mail or messages	Disagree	I am not sure	Somewhat Agree	Agree
g. I feel more free when online	Disagree	I am not sure	Somewhat Agree	Agree
h. I feel that online I can communicate on the same level with others who are older or are more powerful than me	Disagree	I am not sure	Somewhat Agree	Agree
i. I can write insulting things online because there are limited consequences compared to real life	Disagree	I am not sure	Somewhat Agree	Agree
j. I feel like there are less rules when online compared to the real life	Disagree	I am not sure	Somewhat Agree	Agree
k. I feel safer when online compared to real life	Disagree	I am not sure	Somewhat Agree	Agree

8. **Think of your social media account that has the greatest number of followers or friends.** *Please check the answer that best shows how many people on this account you have met in person. Check **only one** box.*
   - ☐ Some of them
   - ☐ Most of them
   - ☐ All of them
   - ☐ I am not sure how many
   - ☐ I don't have a social media account

9. **During the past two months, did you remove any of your followers or friends from your social media account(s) that you had NOT met in person?** *Check **only one** box.*
   - ☐ Yes
   - ☐ No

10. **During the past two months, did you chat or communicate with friends or individuals on social media whom you have NEVER MET in person?** *Check **only one** box.*
    - ☐ Never
    - ☐ Sometimes
    - ☐ Often
    - ☐ I don't have a social media account

11. **During the past two months, when you posted on social media, how often did you mention or tag the town you live in, your school's name, your location, or other personal information?** *Check **only one** box.*
    - ☐ Never
    - ☐ Sometimes
    - ☐ Often
    - ☐ I don't have a social media account

12. **To the best of your knowledge, do your friends chat/ communicate with individuals on social media whom they have never met in person?** *Check **only one** box.*
    - ☐ Never
    - ☐ Sometimes
    - ☐ Often
    - ☐ I don't know

13. **Do you play video games with other online players? *(XBox, PlayStation, PC gaming, etc.)*** *Check **only one** box.*
    - ☐ Never
    - ☐ Sometimes
    - ☐ Often

14. **During the past two months, did you chat/ communicate with people that you didn't know while gaming?** *Check **only one** box.*
    - ☐ Never
    - ☐ Sometimes
    - ☐ Often
    - ☐ I do not play video games

15. Do your parents/guardians ever ask you about what you are doing online?
*Check **only one** box.*

- ☐ I am never online
- ☐ My parents/guardians **do not** ask me about what I do online
- ☐ My parents/guardians **occasionally** ask me about what I do online
- ☐ My parents/guardians **frequently** ask me about what I do online
- ☐ I think my parents/guardians check on my computer and devices to **monitor** what I do online

16. Do your parents have rules for what you are allowed to do online?
*Check **only one** box.*

- ☐ My parents do not have rules for what I can do online
- ☐ My parents have a few rules, but they do not check to see if I follow them
- ☐ My parents have a few rules, and they check to see if I follow them
- ☐ My parents have many rules, and they check to see if I follow them

17. During the past 2 months, have you experienced the following situation ONLINE? *Check the box if the situation occurred.*

- ☐ Someone trying to convince me of their views against people of a race or ethnicity different from mine

18. Do you feel you have a trusted adult to ask for help in case you come across a situation online that makes you feel uncomfortable? *Check **only one** box.*

- ☐ Yes
- ☐ No
- ☐ Not sure
- ☐ It depends on the situation. Please explain below

---

19. How risky do you think it is for the PERSONAL SAFETY of someone your age to engage in the following online behaviors? *For each statement, indicate how risky you think it is by CIRCLING the appropriate answer.*

Circle only one answer in each row

a. To chat online with a stranger	No Risk	Low Risk	I am not sure	Some Risk	High Risk
b. To follow a thread of sexual images/videos	No Risk	Low Risk	I am not sure	Some Risk	High Risk
c. To follow a thread of images/ videos of violence	No Risk	Low Risk	I am not sure	Some Risk	High Risk
d. To chat online with someone who expresses feelings against people because of their race, ethnicity or beliefs	No Risk	Low Risk	I am not sure	Some Risk	High Risk
e. To chat online with someone who is involved in criminal activity	No Risk	Low Risk	I am not sure	Some Risk	High Risk
f. To chat online with someone your age who says they have a weapon	No Risk	Low Risk	I am not sure	Some Risk	High Risk
g. To chat online with someone who sells drugs or alcohol	No Risk	Low Risk	I am not sure	Some Risk	High Risk

20. Which of the following social media tools do you use? *Check only* ***one box*** *for each social media tool.*

Social Media Tool	How often do you use the social media tool?
**Twitter**	☐ All the time<br>☐ Several times a day<br>☐ 1-2 times per day<br>☐ Several times per week<br>☐ Several times per month<br>☐ Never
**Facebook**	☐ All the time<br>☐ Several times a day<br>☐ 1-2 times per day<br>☐ Several times per week<br>☐ Several times per month<br>☐ Never
**Google+**	☐ All the time<br>☐ Several times a day<br>☐ 1-2 times per day<br>☐ Several times per week<br>☐ Several times per month<br>☐ Never
**Houseparty**	☐ All the time<br>☐ Several times a day<br>☐ 1-2 times per day<br>☐ Several times per week<br>☐ Several times per month<br>☐ Never
**YouTube**	☐ All the time<br>☐ Several times a day<br>☐ 1-2 times per day<br>☐ Several times per week<br>☐ Several times per month<br>☐ Never
**WhatsApp**	☐ All the time<br>☐ Several times a day<br>☐ 1-2 times per day<br>☐ Several times per week<br>☐ Several times per month<br>☐ Never
**Skype**	☐ All the time<br>☐ Several times a day<br>☐ 1-2 times per day<br>☐ Several times per week<br>☐ Several times per month<br>☐ Never

**VSCO**	☐ All the time<br>☐ Several times a day<br>☐ 1-2 times per day<br>☐ Several times per week<br>☐ Several times per month<br>☐ Never
**Flickr**	☐ All the time<br>☐ Several times a day<br>☐ 1-2 times per day<br>☐ Several times per week<br>☐ Several times per month<br>☐ Never
**Kik**	☐ All the time<br>☐ Several times a day<br>☐ 1-2 times per day<br>☐ Several times per week<br>☐ Several times per month<br>☐ Never
**Instagram**	☐ All the time<br>☐ Several times a day<br>☐ 1-2 times per day<br>☐ Several times per week<br>☐ Several times per month<br>☐ Never
**Pinterest**	☐ All the time<br>☐ Several times a day<br>☐ 1-2 times per day<br>☐ Several times per week<br>☐ Several times per month<br>☐ Never
**Snapchat**	☐ All the time<br>☐ Several times a day<br>☐ 1-2 times per day<br>☐ Several times per week<br>☐ Several times per month<br>☐ Never
**TikTok**	☐ All the time<br>☐ Several times a day<br>☐ 1-2 times per day<br>☐ Several times per week<br>☐ Several times per month<br>☐ Never

I use other social media tool(s). *Please list up to three, or skip if you do not use other social media tools.*

Please enter the name below:<br>____________________	☐ All the time<br>☐ Several times a day<br>☐ 1-2 times per day<br>☐ Several times per week<br>☐ Several times per month<br>☐ Never
Please enter the name below:<br>____________________	☐ All the time<br>☐ Several times a day<br>☐ 1-2 times per day<br>☐ Several times per week<br>☐ Several times per month<br>☐ Never
Please enter the name below:	☐ All the time<br>☐ Several times a day<br>☐ 1-2 times per day<br>☐ Several times per week<br>☐ Several times per month<br>☐ Never

21. **During the past two months, how frequently did you come across insulting verbal or written expressions against a specific group because of their race, religion, disability, sexual orientation, ethnicity, gender, or gender identity?** *Check **only one** box.*
    - ☐ Very frequently
    - ☐ Frequently
    - ☐ Occasionally
    - ☐ Rarely
    - ☐ Very rarely
    - ☐ Never

22. **During the past two months, where did you come across these insulting expression(s)?** *Check **all boxes** that apply – multiple answers are allowed.*
    - ☐ Verbal speech from a stranger
    - ☐ Verbal speech from a family member
    - ☐ Verbal speech from a person I know (not a family member)
    - ☐ Verbal speech from a teacher or school staff
    - ☐ Poster or flyer on a wall
    - ☐ Spray painting on a wall
    - ☐ Social media
    - ☐ Website
    - ☐ TV
    - ☐ Radio
    - ☐ Music
    - ☐ Book, newspaper, or magazine
    - ☐ I did not come across insulting expressions
    - ☐ Other (please specify below)

____
